# The Impact of Kefir on Epidermal Water Homeostasis in Healthy Human Skin

**DOI:** 10.3390/life12071075

**Published:** 2022-07-19

**Authors:** Emília Alves, João Gregório, Patrícia Rijo, Catarina Rosado, Luís Monteiro Rodrigues

**Affiliations:** 1 CBIOS—Universidade Lusófona’s Research Center for Biosciences and Health Technologies, Campo Grande 376, 1749-024 Lisboa, Portugal; emilia.alves@ulusofona.pt (E.A.); joao.gregorio@ulusofona.pt (J.G.); patricia.rijo@ulusofona.pt (P.R.); catarina.rosado@ulusofona.pt (C.R.); 2 Department of Biomedical Sciences, Faculty of Pharmacy, University of Alcalá, Carretera Madrid-Barcelona, Km 33.100, 28805 Alcalá de Henares, Spain; 3 Instituto de Investigação do Medicamento (iMed.ULisboa), Faculdade de Farmácia, Universidade de Lisboa, 1649-003 Lisboa, Portugal

**Keywords:** kefir, probiotic, skin, water balance, TEWL, epidermal homeostasis

## Abstract

Kefir, a symbiotic consortium of diverse bacteria and yeasts, is one of the most popular probiotic foods on the market. Its consumption has been referred to as beneficial in human skin health, namely in the reinforcement of skin’s barrier function. This benefit likely results from the productive activity of lactic acid bacteria during kefir fermentation. Lactic acid is naturally present in the skin, and actively contributes to epidermal water dynamics and “barrier.” Few studies have been conducted regarding the impact of probiotic consumption in human epidermal water homeostasis. Therefore, this study was designed to explore the impact of the regular consumption of kefir on the skin water dynamics in a group of participants with healthy skin. Participants (n = 27) were healthy female volunteers from whom twelve consumed 100 mL of kefir every day for eight weeks as part of their diet. The remaining (untreated) participants served as the control group. Epidermal water balance was assessed by measuring transepidermal water loss (TEWL) and stratum corneum (SC) hydration on three different occasions—at baseline (T0), after four weeks (T4) and after eight weeks (T8) of interventive kefir consumption. Our study revealed a significant reduction in TEWL (*p* = 0.043) in the kefir group after eight weeks of regular consumption. In the same period, no differences were found for TEWL in the control group (*p* = 0.997). Regarding hydration, skin dryness was progressive in the control group, with a significant reduction in SC hydration (*p* = 0.002) at T8 in comparison to T0. In the kefir group, SC hydration was preserved between T0 and T8 (*p* = 0.997), which we believe to be related to epidermal “barrier” reinforcement. Our study seems to confirm that the regular consumption of kefir does improve cutaneous water balance even in healthy skin.

## 1. Introduction

The complex structure of human skin ensures a protective “barrier” against the penetration of small molecules while preventing the loss of water and electrolytes [1]. It is therefore critical for skin water homeostasis [2,3]. Permeability “barrier” dysfunction, characterized by an increase in transepidermal water loss (TEWL) [1], is typically present in premature infants, but also occurs in later life in many other dermatological conditions involving dry skin [1,2,3]. Kefir, a dietary probiotic currently consumed in many parts of the world as a food supplement [4,5,6], has been referred as potentially beneficial for skin health and in particular to reinforce or restore the skin’s barrier function [7,8,9,10].

Kefir is produced by fermenting milk with kefir grains composed of a symbiotic association of lactic acid bacteria (LAB), acetic acid bacteria and yeast [11,12]. Lactic acid is the principal metabolite of kefir fermentation and is a major component of the surface of human skin, critical for epidermal water balance [13]. Other bioactive compounds produced during kefir fermentation by LAB are hyaluronic acid and sphingomyelinase, both of which seem to be produced in concentrations sufficient to affect the epidermal barrier homeostasis [14]. A clear science-based analysis or demonstration of these properties is still lacking. Most of the studies involving kefir and skin have been developed in vitro and in animal models, focused on potentially beneficial wound healing, anti-inflammatory and antimicrobial activities [15,16,17,18]. However, as we will discuss ahead, a link between the regular consumption of kefir and skin water homeostasis has been suggested, bringing to discussion a new potential interest in this probiotic to prevent xerosis. Our group has been particularly interested in studying the impact of kefir as a food supplement in human skin health [19,20]. Thus, the present study was designed to explore the impact of the regular consumption of kefir on cutaneous water homeostasis in a group of healthy participants, following a case–control design.

## 2. Materials and Methods

### 2.1. Study Design and Intervention

A quasi-experimental study, with a non-equivalent group design, was conducted according to the principles of the Helsinki Declaration and respective amendments [21]. The study protocol was previously approved by the institutional ethics committee (EC.ECTS-P01.18, June 2018). Participants (n = 27) were female, all healthy adults with no history of skin disease or under any kind of regular medication or supplement. By choosing only to include female participants in this preliminary study, we avoided confounding effects related to sex. Participants were recruited by convenience sampling at the university campus following the general inclusion criteria of being aged between 18 and 64 years old. General non-inclusion criteria are listed in Table 1.

After inclusion, physical and socio-demographic data were collected for participant characterization (Table 2). The equivalency of the dietary intake in each participant was confirmed through a three-day dietary record (two weekdays and one weekend day) (available at [19]). Planned experimental procedures were explained and detailed instructions were provided to all individuals. Participants were not restricted from consuming other dairy products, except fermented ones. Important recommendations included keeping their regular eating habits, avoiding over-exercise, not consuming dietary supplements or (other) fermented foods, refraining from using any medications and maintaining their regular skin care and hygiene habits. Participants were instructed not to apply any cosmetic products in the test areas 48 h prior to measurements.

Each participant was allowed to choose either the experimental group, the Kefir Group, or the Control group. In any case, participants were restricted from consuming other fermented foods. The Kefir group included 12 females, who received a 100 mL portion of kefir for consumption daily for eight consecutive weeks. The control group (n = 15) was instructed to not consume any probiotic during the same period.

The general characterization of these participants is summarized in Table 2.

### 2.2. Kefir

The kefir was produced by our team, in our lab, by fermentation of a commercial ultra-high-temperature pasteurized (UHT) semi-skimmed (1.6% fat) cow milk of Portuguese provenance (Nova Açores^®^, S. Miguel, Portugal), with CIDCA AGK1 kefir grains (from Centro de Investigación y Desarrollo en Criotecnología de Alimentos CIDCA, Universidad de La Plata, Argentina) using a grain inoculum of 10% (*w*/*v*), for 24 h, at rest, without shaking, in an open container, at a temperature of 20 ± 1 °C. Activation of the grains, fermentation conditions, and nutritional and microbiological characteristics of kefir are described elsewhere [21]. All participants from the experimental group received a 100 mL portion of kefir every day during the study period.

### 2.3. Biometrics and Experimental Design

Epidermal water balance was assessed by non-invasive methods, which included the quantification of TEWL and stratum corneum (SC) hydration [22]. TEWL is regarded as a measure of the rate of water lost through the skin, reflecting the epidermal “barrier” against desiccation [1,22,23]. TEWL was measured by a Tewameter^®^ TM300 (Courage + Khazaka Electronic GmbH, Köln, Germany) in accordance with published and recently reviewed guidelines [24] and measurements were expressed as g/m^2^/h. The SC water content can also be assessed by other electrometric methods [22,24]. Epidermal hydration was measured by a Corneometer^®^ CM825 (Courage + Khazaka Electronic GmbH, Köln, Germany), a technology based on the detection of skin “capacitance” expressed in arbitrary units (AU) [25]. This study was conducted in Lisbon, Portugal from October to December, which corresponds to the autumn cooling season before winter.

The ventral aspect of the forearm was used for study measurements. Both forearms were marked and referenced for measurements—10 cm below the inner crease of the elbow, and sites randomly chosen [26,27,28]. Measurements were made at baseline (T0), after four (T4) and after eight weeks (T8) of interventive kefir consumption. The same researcher, using identical standards, performed all measurements under controlled temperature (21 ± 1 °C) and humidity conditions (relative humidity, 50 ± 10%) after a period of acclimatization of 20 min.

### 2.4. Statistical Analysis

Results were expressed as the mean ± standard deviation (SD), and as relative frequencies. Normality of data distribution was assessed by the Shapiro–Wilk test. According to the results of the Shapiro–Wilk test, parametric or non-parametric tests were chosen to test different hypotheses. The Chi-Square test was used to test associations between categorical variables. For continuous variables here reported (TEWL and SC hydration), differences within individuals were identified by repeated-measures ANOVA with the Scheffe test for post hoc correction. All analyses were performed using the SPSS statistical package version 25 (SPSS Inc., Chicago, IL, USA) and Jamovi version 2.2 (Sydney, Australia) with a significance level of 0.05.

## 3. Results

As shown in Table 3, no differences were observed at baseline between individuals included in both groups. TEWL and SC hydration values measured at T0, T4 and at T8, in both kefir and control groups, are summarized in Table 3.

**Figure 1:**
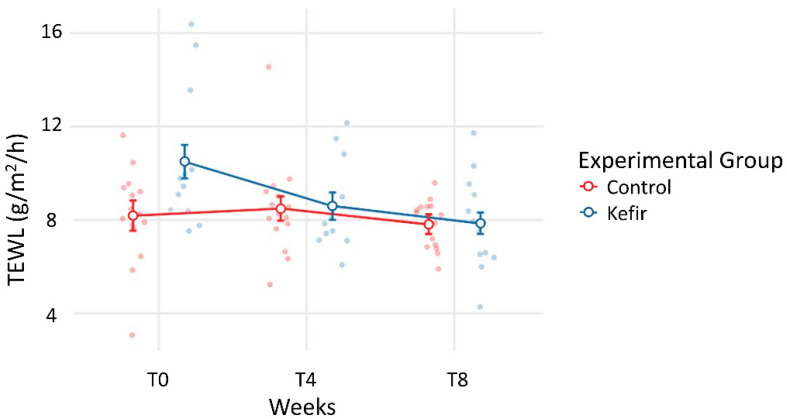
Trends in the mean TEWL recorded for both groups throughout the experimental procedure, between baseline (T0), intermediate period (T4) and end of intervention (T8).

**Figure 2:**
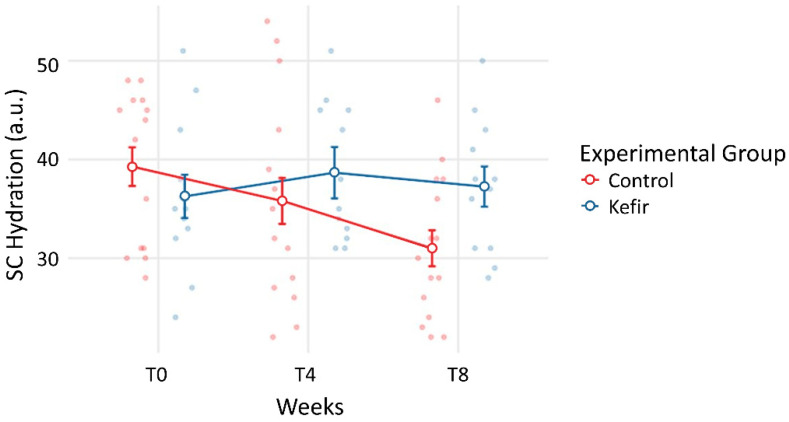
Trends in the mean SC hydration for both groups throughout the experimental procedure, between baseline (T0), the mid-period (T4), and the end of the intervention (T8).

No statistically significant differences in TEWL and SC hydration could be found between kefir and control groups during the experimental procedure. This was expected, as both variables are subject to change due to atmospheric conditions, and reflect participants’ adaptations over time. However, assessing the individual trend of these indicators during the study period, a reduction in TEWL was noticed in the kefir group (Figure 1). This reduction was significant (*p* = 0.043) after eight weeks of intervention (Table 4). By contrast, no significant differences were found for TEWL for the control group in the same period. A tendency toward increased hydration was observed in the kefir group, although the change was not significant (Figure 2). However, SC hydration was markedly reduced in the control group over the same period, significantly different in T8 (*p* = 0.002) compared to T0 (Table 4 and Figure 2).

## 4. Discussion

The present study was designed to look deeper into the alleged impact of kefir regular consumption in cutaneous water balance in healthy individuals. Thus, exploring this new potential property to prevent skin dryness and related xerotic conditions, very common from birth to old age. This study was, therefore, assessed in a healthy population.

Our analysis identified a significant reduction in TEWL after the inclusion of kefir in the regular diet of the intervention group for eight weeks (Table 3 and Table 4, Figure 1) [27]. No other relationships with age or phototype could be found. No differences in TEWL could be identified in the same conditions for the control group consuming no kefir when compared to T0. The oral consumption of probiotics has been referred to be capable of adding important metabolites impacting microbiota [7,10,29,30,31] with potentially beneficial skin effects [14,32,33]. It is the case of lactic and hyaluronic acids resulting from kefir’s fermentation processes [14,34,35,36,37,38] which are also main components of human epidermis, directly involved in the “barrier” preservation [14]. Several mechanisms have been proposed to explain these effects, including improvements of cell adhesion [39], mucin production [39], modulation of the immune system [40], enzymatic activity [41], production of short chain fatty acids and production of organic acids [9,42]. Certainly, lactic acid plays a major role since, as part of the natural moisturizing factor (NMF), it contributes to moisture retention in the skin and also has the ability to enhance the production of ceramides, thus improving the stratum corneum barrier function [14,23].

These outcomes are aligned with the work of Saito et al. who also tested the effects of ingestion of a probiotic strain on the skin of healthy women. Saito et al. only found a decrease in TEWL in the arm, not detecting changes in skin hydration [43] likely related with an eventual restriction of unfavorable intestinal bacterial population. A significant decrease in TEWL was also observed after eight weeks of probiotic intervention with a single strain of *Lactobacillus* species [44]. Another similar study using one *Lactobacillus* species during 12 weeks revealed a significant decrease in TEWL and an increase in SC hydration [45,46]. Beneficial effects on epidermal hydration were also reported elsewhere [47,48]. In our experimental conditions, we could not identify these direct effects in epidermal water hydration. However, as shown in Table 3 and Figure 2, during this experimental period, skin dryness was progressive within the control group such that at T8 a significant reduction in SC hydration was detected when compared with T0. This effect is consistent with the climate conditions registered in this time of the year with lower temperatures and reduced precipitation. By opposition SC hydration differences between T0 and T8 could both be found in the kefir group, likely related with the epidermal “barrier” reinforcement (Table 3, Figure 1).

Under these conditions, a link between the regular consumption of kefir in the diet and an improvement of the epidermal water homeostasis seems to exist as previously suggested [10,32,49]. Nevertheless, the exploratory nature of our study involves some limitations (a) the lack of placebo control, due to the difficulty of using a dairy product without probiotic properties, but excluding milk due to its known effects on the skin [50]; (b) this study was not blinded, since no masking of the product was made, which may represent a bias source; (c) the self-assignment to experimental groups which introduces evitable bias; (d) although developed in humans, all measurements were taken from one single anatomical site. Considering the complexity and diversity of the human epidermal “barrier” in different anatomical sites, a wider mapping of these effects will be beneficial, and potentially complemented with other approaches [51]. In the near future, this line of research must consider studies of longer duration depending on the particular nature of the study and population to be studied.

## 5. Conclusions

Under the present experimental conditions, the regular consumption of kefir seems to improve the epidermal “barrier” and contribute to cutaneous water homeostasis even in the absence of disease. These observations reinforce interest in the use of kefir in cutaneous health.

## Figures and Tables

**Table 1:** General non-inclusion criteria.

Regular consumption of kefir or any probiotic strains (as supplements or pharmaceuticals) in the 3 months prior to this study or during this study Pregnancy or breastfeeding Oncologic disease Chronic illness involving taking regular (daily) medications such as insulin, oral antidiabetics, anti-inflammatories or immunosuppressants Retinoid treatment in the 3 months prior to this study or during this study Topical treatment with corticosteroids/anti-inflammatories in the study area in the 8 days prior to this study or during this study Antibiotic treatment in the 30 days prior to this study or during this study Skin features (naevus, macula, other) in the study areas Cosmetics use involving scrubbing, or depilation at the study areas in the 30 days, or during the study period Failure to comply with the guidelines of this study

**Table 2:** Characteristics of the main study participants (relative frequency (%); mean ± SD).

	Kefir Group (n = 12)	Control Group (n = 15)	*p*-Value
Age, mean (SD), years	29.0 (13.6)	23.8 (6.39)	0.461 ¥
Skin Phototype			0.361
Type II, n (%)	6 (50.0)	5 (33.3)	
Type III, n (%)	4 (33.3)	9 (60.0)	
Type IV n (%)	2 (16.7)	1 (6.70)	
BMI, mean (SD), kg/m^2^	22.1 (3.39)	22.1 (2.91)	0.661 ¥
Education (highest)			0.238
Graduate, n (%)	11 (91.7)	13 (86.7)	
Master, n (%)	0	2 (13.3)	
Doctorate, n (%)	1 (8.30)	0	
Career			0.255
Employed, n (%)	1 (8.30)	0	
University student, n (%)	11 (91.7)	15 (100)	
Residence area			0.076
Urban, n (%)	8 (66.7)	14 (93.3)	
Rural, n (%)	4 (33.3)	1 (6.70)	
Smoking habits			0.053
Smoker, n (%)	3 (25.0)	0	
Occasional smoker, n (%)	1 (8.30)	0	
Non-smoker, n (%)	8 (66.7)	15 (100)	
Alcohol consumption			0.522
Never, n (%)	5 (41.7)	7 (46.7)	
1 to 2 times/week, n (%)	6 (50.0)	8 (53.3)	
3 to 6 times/week, n (%)	1 (8.30)	0	

SD—standard deviation; HK—healthy skin with kefir intake; H0—healthy skin without kefir intake. Groups comparison were made using the Chi-Square test, except (¥) where the Mann–Whitney U test was applied, with *p* < 0.05 for statistical significance.

**Table 3:** Skin measurements (mean ± SD) in kefir (n = 12) and control (n = 15) groups, at baseline (T0), four weeks (T4) and eight weeks (T8) after initiating the intervention. Values were compared by the repeated-measures ANOVA with the Scheffe test for post hoc correction.

	T0			T4			T8		
	Kefir (n = 12)	Control (n = 15)	*p*-Value	Kefir (n = 12)	Control (n = 15)	*p*-Value	Kefir (n = 12)	Control (n = 15)	*p*-Value
TEWL (g/m^2^/h)	10.49 ± 2.98	8.18 ± 2.02	0.361	8.59 ± 1.91	8.49 ± 2.08	1.000	7.85 ± 2.08	7.81 ± 1.04	1.000
SC Hydration (a.u.)	36.25 ± 7.71	39.27 ± 7.49	0.955	38.67 ± 6.97	35.80 ± 10.32	0.983	37.25 ± 6.77	31.00 ± 7.32	0.417

TEWL—transepidermal water loss. SC—stratum corneum hydration. a.u.—arbitrary units.

**Table 4:** Individual mean variation in epidermal water variables between the baseline (T0), the mid-period (T4), and the end of the intervention (T8). Values were compared by the repeated-measures ANOVA with the Scheffe test for post hoc correction (* *p* < 0.05).

	Kefir Group (n = 12)		Control Group (n = 15)	
	T0–T4 (*p*)	T0–T8 (*p*)	T0–T4 (*p*)	T0–T8 (*p*)
TEWL (g/m^2^/h)	1.907 (0.311)	2.643 (0.043) *	−0.305 (0.999)	0.369 (0.997)
Hydration (a.u.)	−2.417 (0.931)	−1.000 (0.997)	3.467 (0.650)	8.526 (0.002) *

TEWL—transepidermal water loss. a.u. arbitrary units.
